# A Family with Atypical Hailey Hailey Disease- Is There More to the Underlying Genetics than ATP2C1?

**DOI:** 10.1371/journal.pone.0121253

**Published:** 2015-04-02

**Authors:** Nina van Beek, Aikaterini Patsatsi, Yask Gupta, Steffen Möller, Miriam Freitag, Susanne Lemcke, Andreas Recke, Detlef Zillikens, Enno Schmidt, Saleh Ibrahim

**Affiliations:** 1 Department of Dermatology, Allergology and Venerology, University of Luebeck, Luebeck, Germany; 2 2nd Dermatology Department, Aristotle University School of Medicine, Papageorgiou General Hospital, Thessaloniki, Greece; Ohio State University Medical Center, UNITED STATES

## Abstract

The autosomal dominant Hailey Hailey disease (HHD) is caused by mutations in the *ATP2C1* gene encoding for human secretory pathway Ca2+/Mn2+ ATPase protein (hSPCA1) in the Golgi apparatus. Clinically, HHD presents with erosions and hyperkeratosis predominantly in the intertrigines. Here we report an exome next generation sequencing (NGS) based analysis of ATPase genes in a Greek family with 3 HHD patients presenting with clinically atypical lesions mainly localized on the neck and shoulders. By NGS of one HHD-patient and *in silico* SNP calling and SNP filtering we identified a SNP in the expected *ATP2C1* gene and SNPs in further ATPase genes. Verification in all 3 affected family members revealed a heterozygous frameshift deletion at position 2355_2358 in exon 24 of *ATP2C1* in all three patients. 7 additional SNPs in 4 ATPase genes (*ATP9B*, *ATP11A*, *ATP2B3* and *ATP13A5*) were identified. The SNPs rs138177421 in the *ATP9B* gene and rs2280268 in the *ATP13A5* gene were detected in all 3 affected, but not in 2 non affected family members. The SNPs in the *ATP2B3* and *ATP11A* gene as well as further SNPs in the *ATP13A5* gene could not be confirmed in all affected family members. One may speculate that besides the level of functional hSPCA1 protein, levels of other ATPase proteins may influence expressivity of the disease and might also contribute, as in this case, to atypical presentations.

## Introduction

The autosomal dominant inherited Hailey Hailey disease (HHD; also benign familiar chronic pemphigus) is caused by mutations (permanent changes of the nucleotide sequence which may affect one or several nucleotides) in the human secretory pathway Ca2+/Mn2+ ATPase protein (hSPCA1) in the Golgi apparatus encoded by the *ATP2C1* gene on chromosome 3q21[1,2]. The *ATP2C1* gene spans 28 exons and has four splice variants, ATP2C1a–d. A variety of more than eighty mutations lead to dysfunction of the protein [1–3]. In this context, impaired calcium levels in the Golgi and cytoplasm have been described [1].

Clinically, HHD presents with erosions and hyperkeratosis predominantly in the intertrigines. Erosions are caused by intraepidermal acantholysis due to a retraction of keratin filaments from the desmosomal plaque and formation of perinuclear aggregates [4]. Disease onset lies around puberty up to 30–40 years with a complete penetrance but variable expressivity. Heat, sweating, mechanical trauma, infections, and UVB exposure can cause exacerbations. Remissions and relapses characterize the course of HHD[5]. Interestingly, extracutaneous manifestations have not been reported despite the ubiquitous expression of hSPCA1 [5].

Several mechanisms have been suggested to explain how a mutation in the *ATP2C1* gene causes blistering in HHD. Most are linked to calcium homeostasis, since it is essential for trafficking of desmosomal proteins to the cell membrane. Calcium and manganese are also required for correct processing, production, and maturation of proteins including glycosylation [6]. Indeed, an overall impaired calcium homeostasis with high resting cytosolic and low Golgi-calcium levels has been shown in keratinocytes of HHD patients [1][7]. In addition, HHD epidermis displayed an altered calcium gradient in vivo [7] which together with abnormal ATP receptor expression may contribute to the blistering [8].

**Fig 1:**
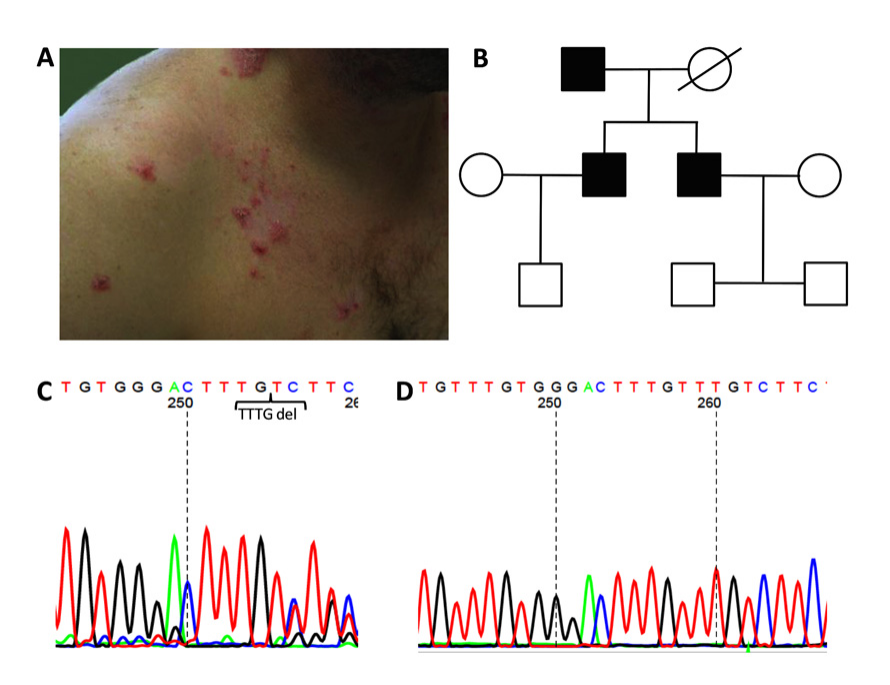
Clinical picture, pedigree, and electropherogram of the Hailey-Hailey disease (HHD) family. Erythematous slightly scaling vesicles, papules, and plaques on the right breast, shoulder, and neck of HHD-affected 1 (**a**). Pedigree of the HHD family. Black squares represent affected males (aged 70, 39 and 35 years), white squares unaffected males (aged 4, 9 and 9 years), white circles represent unaffected women, icons with a crossing line represent family members that had passed away (**b**). ATP2C1 mutation of HHD-affected 2 shown by electropherogram at position 2355_2358 in exon 24 resulting in subsequent frameshift displayed by heterozygous mismatch (**c**) Electropherogram of the unaffected HHD- healthy 1displaying the reference allele (**d**).

In HHD the majority of patients present with crusted erosions and warty papules on skin folds, mainly the axilla, inguinal folds and groin. Interestingly, a family of Greek origin was presenting with clinically atypical HHD, in which lesions were mainly localized on the non-folded skin of neck and the chest, back and shoulders. All lesions first presented around the 25^th^ year of age in all affected family members and had a tendency to recur during the summer months or after sweating and to heal with postinflammatory pigmentation during the cold months of the year. (Fig 1a). Since *ATP2C1* is a member of a large family of genes involved in ATP-dependent ion transport and other members of the ATPase gene family are also involved in calcium homeostasis and desmosomal processing, e.g. *ATP2A2*, the Darier‘s disease gene [3], all ATPase genes in 5 males (3 affected and 2 non-affected) of this family were investigated for single nucleotide polymorphisms (SNPs) by next generation sequencing (NGS).

## Materials and Methods

Blood was drawn from all 5 family members and non-lesional skin of one affected male was obtained after informed consent following the declarations of Helsinki. The participants provided their verbal and written consent and our group has obtained an approval from the ethics committee of the University of Lübeck to conduct genome wide association studies and genomic sequencing of pemphigus patients (approval number 08–156).

Sequence data of one HHD patient gained by NGS Exome Sequencing with Nimblegen Exon array, depth 50x, paired end 100bp reads (BGI, Shenzhen, China) were analyzed for quality, assembled and aligned to the human genome reference build HG19. *In silico* SNP calling and SNP filtering by I) per base quality score II) Phred score, III) 1,000 genomes project frequency (version April 2012_ALL), IV) AV-SIFT Score <0.05 and V) non-synonymous predicted effects identified 66 SNPs, including a deletion in the expected *ATP2C1* gene. Verification by conventional Sanger- resequencing in this patient and two further affected and two unaffected family members was carried out after DNA extraction with Qiagen DNA mini Kit (Qiagen, Hilden, Germany) and PCR using Phusion High- fidelity PCR Kit (New England Biolabs, Germany) following the manufactures instructions (S1 Table). Sanger sequencing was performed at Beckman Coulter Genomics (Essex, United Kingdom). SNP confirmation analysis was performed with Chromas Lite and GENtle.

## Results and Discussion

The SNP verification revealed a heterozygous frameshift deletion at position 2355_2358 in exon 24 of *ATP2C1* in all three patients. This mutation resembles a mutation reported earlier [1,2] and could not be detected in 2 unaffected family members. Interestingly, 7 additional SNPs in 4 ATPase genes, *ATP9B*, *ATP11A*, *ATP2B3* and *ATP13A5*, were identified and confirmed in various family members (Table 1).

**Table 1:** ATPase genes in 3 HHD patients and 2 unaffected family members where SNPs could be verified to a variable extend.

Gene	ref	Observed by NGS	dbSNP 135	Verification by Sanger sequencing					
				affected 1	affected 2	affected 3	healthy 1	healthy 2	affected 2/ skin
**ATP2C1**	TTTG	heterozygous frameshift deletion TTTG	n.a.	TTTG/0	TTTG/0	TTTG/0	TTTG/ TTTG	TTTG/ TTTG	TTTG/0
**ATP11A**	G	heterozygous A/G	rs61746637	G/G	A/G	A/G	A/G	A/G	A/G
**ATP9B**	A	heterozygous A/T	rs138177421	A/T	A/T	A/T	A/A	A/A	A/T
**ATP2B3**	C	homozygous T	n.a.	C/C	T/T	C/C	C/C	C/C	T/T
**ATP13A5Snp1**	A	heterozygous G/A	rs2271791	G/A	G/A	G/A	G/A	G/A	G/A
**ATP13A5Snp2**	C	heterozygous C/T	rs2280268	C/T	C/T	C/T	T/ T	T/T	C/T
**ATP13A5Snp3**	C	heterozygous C/G	rs6797429	C/C	C/G	C/G	C/C	C/C	C/G
**ATP13A5Snp4**	G	heterozygous G/T	rs12637558	T/T	G/T	G/T	G/T	G/T	G/T

ref = reference allele, NGS = exome next generation sequencing performed on affected 2, dbSNP135 = dbSNP database release 135, skin = non-lesional skin of affected 2, n.a. = not available

Remarkably, in the *ATP9B* gene, a heterozygous SNP rs138177421 was detected in all 3 affected, but not in the 2 non affected family members. This SNP is highly conserved and infrequent in populations e.g. 1000genome project frequency 0.0009 (see Table 2). Although little is known about ATP9B, it has recently been identified as a class 2 P4-ATPase, a putative phospholipid flippase translocating phospholipids to the cytoplasm, that localizes to the trans- Golgi network [9]. This type of ATPase is involved in transport vesicle formation and cell polarity by pumping an unknown ion [9]. These alterations in cell polarity might add on to the compromised hSPCA1 function in HHD during Golgi stress. Golgi- and ER- stress have been discussed as being involved in desmosomal processing [8]. Indeed, desmosomal proteins are in particular susceptible to cleavage during apoptosis. Thus, altered cell polarity due to a mutation in *ATP9B* may further challenge the Golgi stress response towards apoptosis resulting in acantholysis.

**Table 2:** Conservation scores and frequencies of SNPs in ATPase genes found in a Greek Family with atypical Hailey Hailey disease.

Gene	ExonicFunc	Aminoacid Change	ESP5400_ALL	1000genomes_ALL	dbSNP135	AVSIFT	LJB_PhyloP	LJB_SIFT	LJB_PolyPhen2
ATP2C1	frameshift deletion	NM_001199182:c.2355_2358del:p.785_786del	NA	NA	NA	NA	NA	NA	NA
ATP13A5	nonsynonymous SNV	NM_198505:c.T3392C:p.V1131A	0.368935	0.36	rs2271791	0.16	0.998904	0.99	35
ATP13A5	nonsynonymous SNV	NM_198505:c.G2215A:p.G739S	0.538855	0.55	rs2280268	0.53	0.857449	0.56	0.0030
ATP13A5	nonsynonymous SNV	NM_198505:c.G397C:p.E133Q	0.549080	0.53	rs6797429	0.96	0.99508	0.4	0.0
ATP13A5	nonsynonymous SNV	NM_198505:c.C287A:p.S96Y	0.371537	0.41	rs12637558	0.02	0.992706	0.98	994
ATP11A	nonsynonymous SNV	NM_015205:c.G2743A:p.A915T	0.020450	0.02	rs61746637	0.14	0.999472	0.83	0.682206
ATP9B	nonsynonymous SNV	NM_198531:c.A617T:p.Q206L	0.001952	0.0009	rs138177421	0.23	0.980726	0.63	0.0010
ATP2B3	nonsynonymous SNV	NM_001001344:c.C422T:p.S141L	NA	NA	NA	0.12	0.980682	0.8	0.0

Gene = Gene name for variant, ExonicFunc = synonymous, non-synonymous, indel, etc., SNV = single nucleotide variant, Aminoacid Change = variant change in nucleotide and protein format, ESP5400_ALL = MAF in Exome Sequencing Project dataset (5,400 exomes) for all populations, 1000genomes_ALL = MAF in 1000Genomes February 2012 release, dbSNP135 = RS# from the dbSNP database, AVSIFT = SIFT Pathogenicity score: closer to 0 is more damaging, LJB_PhyloP = Pathogenicity score from dbNSFP: conserved > 0.95, not conserved < 0.95, LJB_SIFT = Pathogenicity score from dbNSFP: tolerated < 0.95, deleterious > 0.95, LJB_PolyPhen2 = Pathogenicity score from dbNSFP: probably damaging > 0.85, possibly damaging 0.85–0.15, benign < 0.15, NA = not available.

Furthermore, we identified a second SNP in ATP13A5 being unique for the affected family members. *ATP13A5* SNP 2 rs2280268 showed a heterozygous state in all affected individuals studied. In contrast, the 2 non-affected family members displayed a homozygous mutation C→T distinct from the reference genome. Further SNPs in the ATP13A5 gene were present in the unaffected family members as well (*ATP13A5* SNP 1 and 4) or not present in all of the affected family members (*ATP13A5* SNP 3) (Table 1). Since these SNPs are common and conserved in the general population (Table 2), only an accumulation of SNPs in this particular gene may contribute to disease susceptibility in HHD. ATP13A5 belongs to the group of P5-ATPases of which the substrate specificity is unknown. However, recent work suggests that these enzymes affect the intracellular level of different cations, as they localize to vacuolar/lysosomal or apical membranes [10]. In *ATP11A*, a SNP was confirmed by Sanger sequencing in all samples tested except for one affected family member analyzed (Table1) and a homozygous SNP of the *ATP2B3* gene occurred in one affected family member and his skin. These results fit the understanding of HHD as a disease in which the loss of function mutation in ATP2C1 on its own is not sufficient to cause the HHD symptoms[11]. The phenotype is strongly influenced by genetic modifiers and environmental triggers[11]. With the identification of ATP9B and ATP13A5 SNP2 we provide two possible novel genetic modifiers in the orchestra of ionic balance in the Golgi apparatus and hSPCA1.

## Conclusion

Taken together although the level of functional hSPCA1 protein in epidermal cells seems critical [6], levels of other ATPase proteins may influence expressivity of Hailey-Hailey disease. Additionally affected ATPase genes might also contribute, as possibly in this case, to the atypical presentation of the disease. Effects of these mutations could further add on Golgi stress and ionic imbalance, thus might be leaving the skin in affected individuals more vulnerable to environmental triggers than in those patients only carrying an *ATP2C1* mutation. Elucidating ATPase SNP frequencies in diseased patients as well as their role in the pathogenesis of HHD and their functional effects requires further population-based studies and functional investigations in the future.

## Supporting Information

S1 Table Location, genomic and protein effect of predicted SNPs by NGS and primers for validation by Sanger sequenzing. PCR conditions: 10μl HF buffer, 1μl dNTPs, 0.5μl phusion polymerase; all Phusion High fidelity Kit; 2.5μl forward and reverse primer (BiomersGmbH, Ulm, Germany), 100ng sample DNA and nuclease free water adding up to a final volume of 50μl; 98°C 1 min, 32 cycles of 98°C 5 sec/ 68°C 20 sec / 72°C 25 sec, 72°C 10 min. Nonsyn. SNV = nonsynonymous single nucleotide variant.
(DOCX)
