# Supplementary material for: A Family with Atypical Hailey Hailey Disease- Is There More to the Underlying Genetics than ATP2C1?
Source: PLoS One. 2015 Apr 2;10(4):e0121253. doi: 10.1371/journal.pone.0121253 (PMC4383578; doi:10.1371/journal.pone.0121253)
Supplement: S1 Table — PCR conditions: 10μl HF buffer, 1μl dNTPs, 0.5μl phusion polymerase; all Phusion High fidelity Kit; 2.5μl forward and reverse primer (BiomersGmbH, Ulm, Germany), 100ng sample DNA and nuclease free water adding up to a final volume of 50μl; 98°C 1 min, 32 cycles of 98°C 5 sec/ 68°C 20 sec / 72°C 25 sec, 72°C 10 min. Nonsyn. SNV = nonsynonymous single nucleotide variant. (DOCX) [file pone.0121253.s001.docx]

**Supplementary Material**

**S1 Table: Location, genomic and protein effect of predicted SNPs by NGS and primers for validation by Sanger sequenzing.**

| Gene | SNPeffect | SNP | Position | Protein change | Primer forward | Primer reverse |
| --- | --- | --- | --- | --- | --- | --- |
| **ATP2C1** | Frameshift deletion | 2355_2358del | Chr3:130716576 | 785_786del | TTTTGCGTAATCAGCCAGCG | AGCTCCCTTAACTTCCCAGC |
| **ATP11A** | Nonsyn. SNV | 2743 G->A | Chr13:113514616 | 915 A->T | CACATGCAACGTGCCCATAG | GTACAGGGTCGGGTCTCTCT |
| **ATP9B** | Nonsyn. SNV | 617 A->T | Chr18:76886325 | 206 Q->L | TTGGGGAGTATACTAAGAGTTGCAT | GCTTGTGTGGTTGTGTGTGC |
| **ATP2B3** | Nonsyn. SNV | 422 C->T | chrX:152807142 | 141 S ->L | ACCAATGACCTGGAGAAGCG | CACCACACAGATGACGGACA |
| **ATP13A5 Snp1** | Nonsyn. SNV | 3392 T->C | Chr3:192994543 | 1131 V-> A | GGGGGATCAAAGTGGTTGCT | CACAGGCCAAGAGGTAGTCC |
| **ATP13A5 Snp2** | Nonsyn. SNV | 2215 G->A | Chr3:193031926 | 739 G ->S | TCCCAGGTCCAGTCTCTTGG | CTTGTTGCTGAGGGGAGGTT |
| **ATP13A5 Snp3** | Nonsyn. SNV | 397 G->C | Chr3:193080414 | 133 E ->Q | AGGATATCTCCGCTGCCAGT | TCCTGCATTGGACACCACTC |
| **ATP13A5 Snp4** | Nonsyn. SNV | 543 A->G | Chr3:193209178 | 181 I-> M | TGGTTTATGACAGAGTGGCGG | CATGAGCAGTGGCTGTCAGT |

PCR conditions: 10µl HF buffer, 1µl dNTPs, 0.5µl phusion polymerase; all Phusion High fidelity Kit; 2.5µl forward and reverse primer (BiomersGmbH, Ulm, Germany), 100ng sample DNA and nuclease free water adding up to a final volume of 50µl; 98°C 1 min, 32 cycles of 98°C 5 sec/ 68°C 20 sec / 72°C 25 sec, 72°C 10 min.

Nonsyn. SNV = nonsynonymous single nucleotide variant
